# Improving the clinical workflow of a MR-Linac by dosimetric evaluation of synthetic CT

**DOI:** 10.3389/fonc.2022.920443

**Published:** 2022-08-29

**Authors:** Bin Tang, Min Liu, Bingjie Wang, Peng Diao, Jie Li, Xi Feng, Fan Wu, Xinghong Yao, Xiongfei Liao, Qing Hou, Lucia Clara Orlandini

**Affiliations:** ^1^ Department of Radiation Oncology, Sichuan Cancer Hospital and Research Institute, affiliated to University of Electronic Science and Technology of China (UESTC), Chengdu, China; ^2^ Key Laboratory of Radiation Physics and Technology of the Ministry of Education, Institute of Nuclear Science and Technology, Sichuan University, Chengdu, China; ^3^ Faculty of Arts and Science, University of Toronto, Toronto, ON, Canada

**Keywords:** synthetic CT, clinical workflow, MR-Linac, dosimetry, radiotherapy

## Abstract

Adaptive radiotherapy performed on the daily magnetic resonance imaging (MRI) is an option to improve the treatment quality. In the adapt-to-shape workflow of 1.5-T MR-Linac, the contours of structures are adjusted on the basis of patient daily MRI, and the adapted plan is recalculated on the MRI-based synthetic computed tomography (syCT) generated by bulk density assignment. Because dosimetric accuracy of this strategy is a priority and requires evaluation, this study aims to explore the usefulness of adding an assessment of dosimetric errors associated with recalculation on syCT to the clinical workflow. Sixty-one patients, with various tumor sites, treated using a 1.5-T MR-Linac were included in this study. In Monaco V5.4, the target and organs at risk (OARs) were contoured, and a reference CT plan that contains information about the outlined contours, their average electron density (ED), and the priority of ED assignment was generated. To evaluate the dosimetric error of syCT caused by the inherent approximation within bulk density assignment, the reference CT plan was recalculated on the syCT obtained from the reference CT by forcing all contoured structures to their mean ED defined on the reference plan. The dose–volume histogram (DVH) and dose distribution of the CT and syCT plan were compared. The causes of dosimetric discrepancies were investigated, and the reference plan was reworked to minimize errors if needed. For 54 patients, gamma analysis of the dose distribution on syCT and CT show a median pass rate of 99.7% and 98.5% with the criteria of 3%/3 mm and 2%/2 mm, respectively. DVH difference of targets and OARs remained less than 1.5% or 1 Gy. For the remaining patients, factors (i.e., inappropriate ED assignments) influenced the dosimetric agreement of the syCT vs. CT reference DVH by up to 21%. The causes of the errors were promptly identified, and the DVH dosimetry was realigned except for two lung treatments for which a significant discrepancy remained. The recalculation on the syCT obtained from the planning CT is a powerful tool to assess and decrease the minimal error committed during the adaptive plan on the MRI-based syCT.

## Introduction

The roles of image guidance, adaptive planning, and magnetic resonance imaging (MRI) in radiation therapy have been increasing over the last two decades. Several authors have recently demonstrated that the possibility to modify the radiation treatment according to the patient’s daily anatomy (1) can effectively manage the inter-fraction variation in OAR and target position and shape that adversely affect treatment accuracy and patient outcomes (2, 3). Most recently, magnetic resonance (MR)–guided radiation therapy (MRgRT) has provided the opportunity for fractional online adaptive radiotherapy (ART) for patients undergoing radiation therapy (4). The hybrid RT machines, combining a MR scanner with a RT delivery system, enable soft tissue contrast daily MRI to visualize all anatomical changes during the course of radiotherapy (5–7); subsequently, the adaptive planning taking into account changes in target/OAR shape and position can be performed.

Several centers presented the feasibility and clinical advantages of using MR-Linear accelerator (MR-Linac) machines for the treatment of tumors located in different sites, and this method is becoming increasingly popular (1, 7–10). Although this clear clinical advantage, there are approximations in the procedure, i.e., the assignment of CT-based electron density (ED) values to structures in the MR image data set, that should be investigated by the user to ensure the quality of the treatment performed (11). Because of the lack of tissue density information needed for dose calculation in MRgRT, an ED map was generated from MRI to allow for adaptive planning based on daily MRI. To address this issue, several approaches have been developed to generate ED maps also called synthetic computed tomography (syCT) from MRI (MRI-based syCT): i) the bulk density assignment (12–19) consisting in a direct density assignment methods that determine volumes of interest (tissues or organs) on the patient’s MRI and assign them a given density; ii) atlas-based method (20–22) based on atlas registration that spatially map an image (for example, CT) to the patient’s MRI, merging them to generate a pseudo CT scan; and iii) voxel-by-voxel conversion (23–27) and deep learning (28–40) are based on statistical learning model relationships between CT and MRI intensities and then apply the resulting model to the patient’s MRI. As an emerging method, deep learning indeed shows promising results, reflecting in high accuracy, automation, and efficiency in the generation of syCT from MRI. However, there are still some obstacles to be overcome before deep learning–based methods can be implemented, for example, most of those studies are anatomical site-specific, MRI sequence–specific, lack of external validation, etc. Bulk density assignment is a straightforward way to generate syCT. As the simplest way of bulk assignment, assigning the whole patient volume to a single homogeneous media could produce a relative large dose error (> 2%) (15, 41); more sophisticated ways, such as separating the tissues in MRI into several classes and then assigning an ED to each class, show clinically acceptable error (around 1%) (17, 42). Previous studies (43, 44) also reveal the concerns regarding the impact of density inhomogeneity on patient dosimetry in the presence of a magnetic field. The strategy of syCT applied in Monaco (Elekta AB, Stockholm, Sweden) treatment planning system (TPS) for 1.5-T MRgRT is the bulk density assignment based on the contours drawn on patient simulation CT. More specifically, during the online adapt-to-shape (ATS) procedure, the daily acquired MRI will be deformably registered to simulation CT, then all contours information including average ED and the priority of density assignment on CT are propagated to MRI, and then syCT is generated by the bulk density assignment on MRI. It is well known that contouring is a critical step in the radiotherapy process (45); the quality of the initial contours can have significant effect on the therapeutic ratio, and several studies have linked contouring protocol deviations with decreased survival and increased toxicity (46). In the case of adapted plans recalculated on syCTs generated by the bulk density assignment of the contours, contouring has a much greater weight as it also impacts on the accuracy of the calculation itself. Considering that the adapted plan is reoptimized and recalculated on the syCT, the dosimetric accuracy of this strategy is a priority and requires evaluation (47–49). In this article, on the basis of our clinical experience, we propose the addition of a simple procedure into the clinical routine to ensure that the dosimetric error of syCT generated by the bulk density assignment remains small.

## Materials and methods

### Patients

Sixty-one patients consecutively treated at 1.5-T MR-Linac (Unity, Elekta, Crawley, UK) were included in this study. Patients who had different tumor sites were treated with different prescription doses and referring physicians. All patients provided informed oral consent at the use of their clinical data for research purposes. The study was approved by the institutional ethics Committee (SCCHEC-02-2022-003).

### CT and MR simulation

Patients were set up in the supine position using indexed patient positioning aids; with the exception of brain treatments and the first two rectum patients for which thermoplastic masks were used, all patients were immobilized using Wing-STEP, KneeSTEP, and FeetSTEP supports (IT-V, Innsbruck, Austria). Patients underwent a three-dimensional (3D) or four-dimensional (4D) (lung and liver cases) CT scans; a first non-contrast CT series used for the treatment planning was acquired, followed by one with the contrast used as support for the physician in the tumor delineation; in addition, T2-weigthed simulation scans on the 1.5-T MR-Linac were acquired immediately after CT simulation in the same position and with the same immobilization devices. Patients with liver and kidney disease were asked to wear a containment belt to reduce the respiratory movement, reducing artifacts as well at the simulation and delivery session. Considering the dosimetric challenge that can result from inconsistent bladder filling between simulation and delivery (50), patients with disease of the rectum, prostate, and cervix treated, prior to CT and MRI simulation scans and then prior to each MRI performed on the day of treatment (upon patient arrival and after approval of the treatment plan prior to beam delivery), were fitted with a bladder catheter to ensure the same bladder filling between the daily MR scan and treatment delivery.

### Delineation and planning on reference CT

The delineation of targets and OARs and the reference treatment planning were performed in Monaco V5.4. The referring physician of each patient contoured on non-contrast CT but with the use of available imaging studies. For moving targets, the motion of the tumor was determined using information from 4D-CT; for liver and lung cases, an internal target volume was created under consideration of the maximum intensity projection (MIP) and minimum intensity projection, generated by reconstructing 10 respiratory phases. A reference plan was then optimized to achieve clinical goals using eight to 12 individual beam angles, 3-mm dose grid, and 1% calculation uncertainty.

### Daily online ART

Daily Online ART was delivered in one of the two available procedures conventionally called ATP (adapt to position) and ATS (5). In both modalities, once the patient is positioned on the couch, an MRI is acquired then rigidly registered to the CT images of the reference plan. At this point, the plan can be adapted, i.e., optimized and recalculated on the reference CT images and contours accordingly to the patient positioning error determined by the image registration; this is the ATP workflow. On the other hand, the ATS workflow will be performed if there is still a large residual error although rigid registration has been applied, mainly because the shape or/and position of certain organ changes compared to when the reference CT was acquired. In ATS workflow, adjusting of contours according to daily MRI is needed, which is one of the most time-consuming (51, 52) steps; therefore, considering that there may be a significant time interval between the acquisition of the first daily MRI and beam delivery, our center while approving the adapted plan performs a second MRI that is rigidly registered with the one performed at the beginning of the session and on which the plan was adapted, to ensure the appropriateness of the ongoing delivered treatment. In case of position differences, a next step is performed, adjusting the isocenter position and recalculating the dose according to the ATP procedure.

### Patient-specific QA

Treatment plan verifications were performed by comparing the x-ray fluence measured by a 3D dosimetry array (ArcCHECK^®^-MR, Sun Nuclear Corp., Melbourne, FL) with that computed by the TPS, for the reference plan and each adapted plan. The global gamma analysis with percentage signal to agreement and distance to agreement of the criteria of 3%/3mm was used for evaluation. The verification of the reference CT plan was performed before the start of the treatment course, whereas the online adapted plans based on the reference CT plan were verified (as only option for the nature of the clinical workflow) after their delivery to check the adequacy of the already administered treatment.

### Dosimetric evaluation of synthetic CT

**Figure 1:**
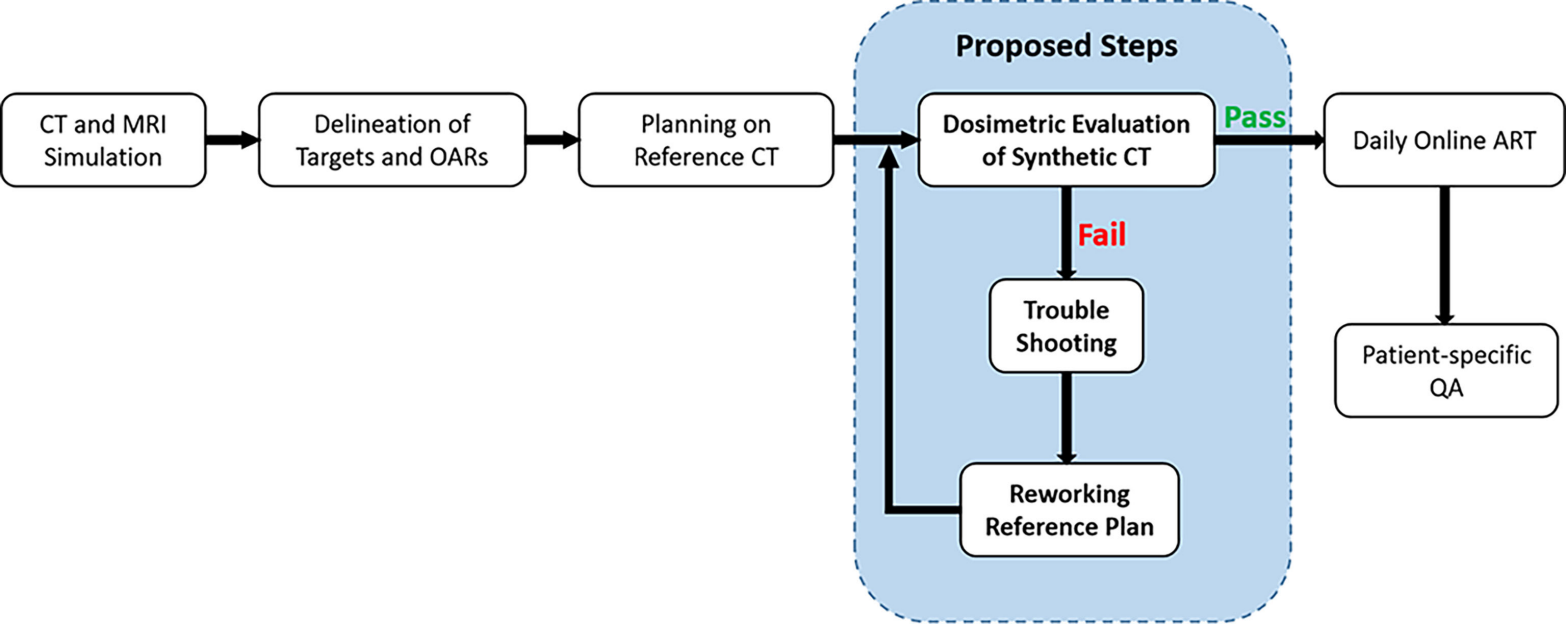
Clinical workflow integrated with quality control on dosimetric accuracy of synthetic CT plan.

The clinical workflow was extended by adding extra steps between treatment planning on the reference CT and daily ART, as shown in **Figure 1**. The ready-to-use reference CT plan in the ATS workflow contains all the density bulk assignment information, i.e., the contours, their corresponding average ED, and the priority of each contour concerning density assignment in case of contour overlaps. The reimaging of a specific district of a patient does not result in perfectly superimposable images, as organ profiles and/or their relative positions are influenced by many factors such as bladder and/or rectum filling, gas in the intestines, and contouring. Therefore, to assess the dosimetric error of plan recalculation on syCT caused by the loss of density inhomogeneity during the ATS procedure and to exclude other potential factors affecting the results of the calculation (i.e., recontouring on daily MRI), for each patient, the “ideal” syCT is generated using the reference CT imaging, contours and EDs, and the priorities indicated in the reference plan; then, the reference CT plan is recalculated (not reoptimized) on the generated syCT. Then, targets and organs at risk (OARs) dose–volume histogram (DVH) of the CT and syCT plans as well as the dose distributions using gamma analysis with the criteria of 3%/3 mm and 2%2 mm were compared. The DVHs and corresponding dosimetric parameters of the CT and syCT treatment plans were obtained directly from Monaco TPS. The volume of the target covered by the prescription dose (V_Dpre_) and the clinically concerned OAR dosimetric parameters with the corresponding constraints were compared from the DVHs. DICOM-RT files including CT images, RT plans, RT structures, and RT dose were exported to MATLAB R2013a; 3D gamma analysis between dose of CT and syCT was performed by CERR v4.4 (https://github.com/cerr/CERR). Treatment plan calculation was considered in agreement when the percentage of points with γ< 1 is higher than 99% with the criteria of 3%/3 mm and the target dose difference in any point of the DVH is lower than 1.5% or 1 Gy.

### Trouble shooting and reworking of reference plan

Dose discrepancy could be detected by the comparison of CT vs. syCT DVHs and/or the gamma analysis. The causes were then investigated by several means, including a preliminary visual comparison of the syCT vs. CT and its contours, spotting abnormalities in ED values assignment to the various contours, checking priority assigned in case of their overlap in the treatment plan, focusing on the organs/regions with the greatest deviations, etc.

After the corresponding corrections were made in the reference plan, a new syCT was generated, and the plan was recalculated on the new syCT. Then, the dosimetry of the two plans was compared again. If the causes were not obvious or the dosimetric difference persists, then the discrepancy was notified to the radiation oncologists for their clinical decision regarding treatment delivery.

## Results

The clinical characteristics of the patients and the treatment schedule followed are shown in **Table 1**. Included patient samples were 57.4% of men and 42.6% of women, with a median age of 58 years at the time of radiotherapy; among the investigated treatment sites, the liver was the most common with 21 patients treated, followed by cervix and rectum with 12 and seven cases, respectively; 77.0% of patients followed the ATS workflow and the remainder the ATP workflow.

**Table 1:** Patient characteristics, tumor site, and adopted treatment schedule.

	N°	Age	Sex	BMI	Dose	Dose/fr	Schedule
		year	no.	kg/m^2^	Gy	Gy/fr	no.
		median (range)	M-F	median (range)	range	range	ATS-ATP
**Rectum**	7	50 (45–63)	6–1	23.5 (19.0–29.7)	25	5	7–0
**Cervix**	12	55 (44–66)	0–12	25.9 (20.8–29.4)	10–25	5	12–0
**Lung**	2	64 (58–70)	2–0	21.4 (19.8–23.1)	42–48	6–8	0–2
**Prostate**	3	71.5 (60–83)	3–0	22.2 (19.5–24.9)	56	2.8	3–0
**Kidney**	4	58 (58–69)	3–1	21.5 (20.9–24.2)	36–50	5–8	3–1
**Pancreas**	6	62 (48–78)	3–3	23.9 (20.8–25.4)	36–48	6–8	2–4
**Liver**	21	52 (41–75)	14–7	22.7 (16.4–25)	24–50	6–15	16–5
**Brain**	6	57 (31–68)	4–2	21.7 (16.4–26.7)	36–50	5–8	4–2
**Total**	61	58 (31–5)	35–26	23.4 (16.4–29.4)	10–50	5–15	47–14

BMI, body mass index; ATP, adapt to position; ATS, adapt to shape.

A total of 475 reference and adaptive plan verifications were performed with ArcCHECK. The results of gamma analysis with the criteria of 3%/3 mm for different tumor sites showed high accuracy with a median pass rate of 98.2% (range, 92.3%–100.0%); the details were shown in **Table 2**.

**Table 2:** Quality assurance of treatment plans with ArcCheck: results of gamma analysis of reference and adapted plans.

	Pts no.	Reference plan no.	Adapted plan no.	3%/3 mm (%)
				median (range)
**Rectum**	7	7	35	99.2 (95.6–100.0)
**Cervix**	12	12	43	97.6 (92.3–100.0)
**Lung**	2	2	13	98.3 (95.2–100.0)
**Prostate**	3	3	60	97.5 (93.3–100.0)
**Kidney**	4	4	31	97.2 (95.7–99.6)
**Pancreas**	6	6	36	97.6 (93.0–100.0)
**Liver**	21	21	154	98.8 (93.2–100.0)
**Brain**	6	6	42	97.0 (92.9–100.0)
**Total**	61	61	414	98.2 (92.3–100.0)

The procedure to generate a syCT from the original reference CT and, subsequently, to recalculate the plan on the syCT took an average time of 312 s (minimum, 253 s; maximum, 400 s); it is therefore feasible within the clinical workflow. The dosimetry of the reference CT plan was consistent with the corresponding dosimetry on the syCT for 54 of the 61 patients: The absolute difference of the target and OARs DVH dosimetric parameters remained below 1.5% for ∇*Vx* . (percentage of volume receiving × Gy) and lower than 1 Gy for∇*Dy* . (dose in Gy received by the volume y), respectively, as reported in **Table 3**; similarly, the results of the dose distribution comparison performed on the whole volume, targets, and OARs by the 3D gamma analysis with the criteria of 3%/3 mm and 2%/2 mm were 99.7% and 96.3%, respectively, confirming the agreement found with the DVHs comparison. The details of the results obtained are shown in **Table 4**.

**Table 3:** Absolute difference of CT vs. syCT targets and OARs DVH dosimetric parameters (mean and standard deviations) for 54 out of 61 patients, resulting in tolerance at the first CT vs. syCT plan comparison.

Site (no.)	CT vs. SyCT absolute dosimetric difference for targets and OARs									
**Rectum (5)**	*PTV*	*Bladder*		*Small Bowel*		*Colon*	*Fem Heads*			
	V_Dpre_ (%)	D_0.5cc_ (Gy)	D_10cc_ (%)	D_0.5cc_ (Gy)	D_5cc_ (Gy)	D_0.5cc_ (Gy)	D_max_ (y)	D_10cc_ (Gy)
	0.27 ± 0.33	0.17 ± 0.06	0.09 ± 0.13	0.30 ± 0.23	0.32 ± 0.38	0.35 ± 0.34	0.59 ± 0.28	0.06 ± 0.09
**Prostate (3)**	*PTVs*	*Bladder*		*Rectum*	*Rectum*	*Urethra*	*Fem. Heads*	*Sigmoid*
	V_Dpre_ (%)	D_15%_ (Gy)	D_60%_ (Gy)	D_15%_ (Gy)	D_60%_ (Gy)	D_max_ (Gy)	D_50%_(Gy)	D_2cc_ (Gy)
	0.60 ± 0.53	0.32 ± 0.47	0.05 ± 0.05	0.05 ± 0.01	0.04 ± 0.04	0.05 ± 0.02	0.01 ± 0.01	0.41 ± 0.47
**Cervix (12)**	*PTVs*	*Bladder*		*Rectum*		*Intestine*	*Fem Heads*			
	V_Dpre_ (%)	D_1cc_ (Gy)	D_2cc_ (Gy)	D_1cc_ (Gy)	D_2cc_ (Gy)	D_2cc_ (Gy)	V_10Gy_ (%)	Dmax		
	0.40 ± 0.33	0.08 ± 0.05	0.05 ± 0.05	0.12 ± 0.18	0.25 ± 0.29	0.13 ± 0.11	0.04 ± 0.03	0.16 ± 0.21		
**Pancreas (5)**	*PTVs*	*Spinal cord*	*Intestine*	*Liver*		*Pancreas-PTV*	*Kidney*	Stomach		
	V_Dpre_ (%)	D_max_ (Gy)	D_1cc_ (Gy)	V_20Gy_ (%)	V_5Gy_ (%)	D_33%_ (Gy)	D_20%_ (Gy)	D_1cc_ (Gy)		
	0.37 ± 0.39	0.15 ± 0.5	0.09 ± 0.08	0.13 ± 0.24	0.17 ± 0.31	0.04 ± 0.05	0.01 ± 0.01	0.06 ± 0.09		
**Liver (19)**	*PTVs*	*Spinal cord*	*Duodenum*	*Liver -GTV*		Small bowel	Kidney	Bladder		
	V_Dpre_ (%)	D_max_ (Gy)	D_1cc_ (Gy)	D_mean_ (Gy)	V_5Gy_ (%)	D_0.5cc_ (Gy)	D_mean_ (Gy)	D_1cc_ (Gy)		
	0.44 ± 0.76	0.05 ± 0.34	0.06 ± 0.06	0.01 ± 0.04	0.02 ± 0.08	0.07 ± 0.01	0.02 ± 0.04	0.13 ± 0.10		
**Kidney (4)**	*PTVs*	*Spinal cord*	Intestine	*Liver*	*Kidney-GTV*		*Kidney cL*	*Pancreas*		
	V_Dpre_ (%)	D_max_ (Gy)	D_1cc_ (Gy)	V_5Gy_ (%)	V_10Gy_ (%)	D_max_ (Gy)	D_max_ (Gy)	D_max_ (Gy)		
	0.66 ± 0.39	0.05 ± 0.01	0.20 ± 0.13	0.09 ± 0.11	0.19 ± 0.45	0.10 ± 0.12	0.11 ± 0.06	0.60 ± 0.54		
**Brain (5)**	*PTVs*	*Brain stem*		*Lens*	*Brain*	*Eyes*	*Opt.Chiasm*	*Opt. nerves*		
	V_Dpre_ (%)	D_max_ (Gy)	D_50%_ (Gy)	D_max_ (Gy)	D_mean_ (Gy)	D_max_ (Gy)	D_max_ (Gy)	D_max_ (Gy)		
	0.61 ± 0.73	0.01 ± 0.22	0.02 ± 0.03	0.03 ± 0.04	0.06 ± 0.08	0.10 ± 0.09	0.06 ± 0.06	0.06 ± 0.06		

**Table 4:** Median values and range of gamma analysis results performed comparing the dose distribution on CT to syCT for 54 patients with good agreement at the first check of the CT vs. syCT DVHs dosimetric parameters.

		Body (n = 54)	Targets (n = 69)	OARs (n = 270)
**Gamma analysis**	**3%/3 mm** (%)	99.7(99.0–100.0)	100.0 (98.4–100.0)	100.0 (83.4–100.0)
	**2%/2 mm** (%)	98.5 (95.7–99.8)	96.3 (84.7–100.0)	99.9 (78.1–100)

Seven patients (two lung, two liver, two rectum, and one brain treatments, respectively) presented a discrepancy between CT and syCT target and/or OARs DVH dosimetry as well as lower gamma pass rate. Details of the dosimetric differences and gamma analysis obtained at first screening and after a rework of the reference plan are shown in **Tables 5**, **6**, **7**.

**Table 5:** Absolute difference of target and OARs DVH dosimetric parameters of the original CT and of the reworked CT vs. the corresponding syCT plans.

		PTV		Omolateral Lung		Body	
		Original	Reworked	Original	Reworked	Original	Reworked
Lung Case 1	ΔV_Dpre_ (%)	6.1	6.2	–	–	–	–
	ΔV5Gy (%)		–	1.1	1.1	–	–
	γ (3%/3 mm) (%)	40.7	62.4	86.1	87.4	93.2	93.9
	γ (2%/2 mm) (%)	19.6	33.5	64.5	65.5	82.4	82.4
Lung Case 2	ΔV_Dpre_ (%)	18.6	17.9	–	–	–	–
	ΔV5Gy (%)	–	–	0.7	0.7	–	–
	γ (3%/3 mm) (%)	59.9	59.1	100.0	100.0	98.8	98.8
	γ (2%/2 mm) (%)	31.2	30.1	84.4	85.0	94.9	95.0

OARs not reported in the table presented CT vs. syCT absolute difference of DVHs dosimetric parameters< 1 Gy or 1.5%, and a gamma analysis with 3%/3 mm and 2%/2 mm > 99.0% and 95.0%, respectively.

**Table 6:** Absolute difference of target and OARs DVH dosimetric parameters of the original CT and of the reworked CT vs. the corresponding syCT plans.

		PTV1		PTV2		PTV3		Colon		Body	
		Original	Reworked	Original	Reworked	Original	Reworked	Original	Reworked	Original	Reworked
Rectum Case 1	ΔV_Dpre_ (%)	14.83	0.49	9.12	0.23	8.05	0.06	**-**	**-**	**-**	**-**
	ΔD_20cc_ (Gy)	**-**	**-**	**-**	**-**	**-**	**-**	0.69	0.08	**-**	**-**
	γ (3%/3 mm) (%)	89.98	99.05	93.7	99.03	87.59	99.04	97.91	98.34	97.87	99.32
	γ(2%/2 mm) (%)	51.71	78.85	58.66	78.12	41.76	78.52	89.33	89.93	87.34	94.84
Rectum Case 2	ΔV_Dpre_ (%)	20.99	0.25	5.95	0.42	23.85	0.52	–	–	–	–
	ΔD_0.5cc_ (%)	–	–	–	–	–	–	3.94	0.03	–	–
	γ (3%/3 mm) (%)	79.88	99.64	93.9	99.6	77.32	98.14	82.42	89.10	95.51	99.28
	γ (2%/2 mm) (%)	44.04	83.95	68.87	87.38	40.01	84.05	68.48	78.08	85.31	94.95

OARs not reported in the table presented CT vs. syCT absolute difference of DVHs dosimetric parameters< 1 Gy or 1.5%, and a gamma analysis with 3%/3 mm and 2%/2 mm > 99.0% and 95.0%, respectively.

**Table 7:** Absolute difference of target DVH dosimetric parameters between CT and syCT, before and after plan reworking.

	Parameter	PTV		Body	
		Original	Reworked	Original	Reworked
Brain Case	ΔV_Dpre_ (%)	20.22	1.08	–	**-**
	γ (3%/3 mm) (%)	97.23	99.90	99.68	99.90
	γ (2%/2 mm) (%)	69.87	96.07	94.77	97.64
Liver case 1	ΔV_Dpre_ (%)	8.32	1.30	**-**	**-**
	γ (3%/3 mm) (%)	75.37	99.84	97.26	99.66
	γ (2%/2 mm) (%)	33.51	94.68	92.39	98.33
Liver case 2	ΔV_Dpre_ (%)	4.72	0.5	–	–
	γ (3%/3 mm) (%)	81.97	99.74	97.06	99.53
	γ (2%/2 mm) (%)	60.71	94.31	89.53	96.88

OARs were not reported in the table because they presented CT vs. syCT absolute difference of DVHs dosimetric parameters< 1 Gy or 1.5%, and a gamma analysis with 3%/3 mm and 2%/2 mm > 99.0% and 95.0%, respectively.

**Figure 2:**
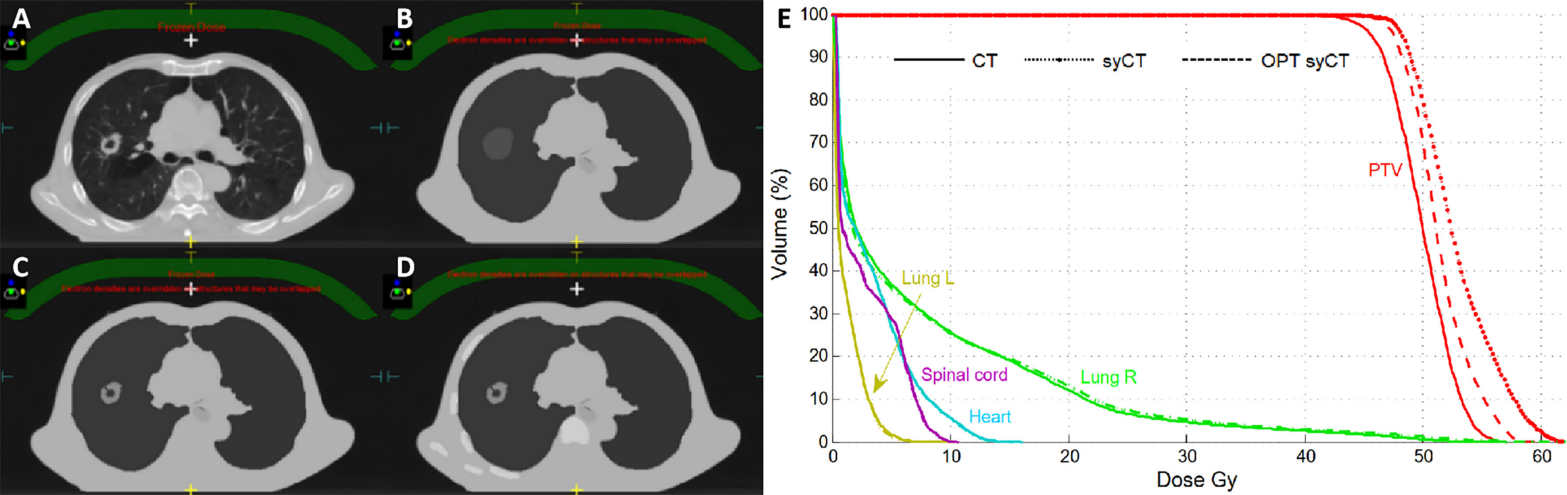
Successive attempts to realign the CT and syCT plan dosimetries for lung case 1. Reference CT representative transversal image **(A)**, corresponding syCT with ED assignments on successive attempts **(B–D)**, and corresponding DVHs **(E)**.

Large dosimetric discrepancies were recorded for both patients with lung cancer with target *V*_Dpre_ . = 6.1% and 18.6% for cases 1 and 2, respectively, persisting even after plan optimization ( *V*_Dpre_ . = 6.2% and 17.9%, respectively). Because of the high ED gradient within the target, and between the target and the remaining part of the lung, the assignment of an average ED was not able to accurately reproduce the dose calculation. **Figure 2** shows the successive unsuccessful attempts made for lung case 1 to decrease the dosimetry discrepancy between syCT and reference CT plan. In the original plan, an average ED was assigned to the whole target drawn by the physician, as can be seen in the corresponding syCT (**Figure 2B**); then, the inhomogeneous density of the target was considered with a precise contour of relative high-density parts (**Figure 2C**); in the last attempt, the bones intercepting the beams entry were drawn (**Figure 2D**). Although there was an improvement in the target gamma analysis, the dosimetric difference was not reduced (**Figure 2E**), and the patient treatment proceeded using the ATP workflow. For lung case 2, the contours were set with appropriate ED in the original CT plan, and subsequent attempts did not lead to improvements; similarly, the patient was treated according to ATP workflow.

For the liver, rectum, and brain treatments, once the source of the errors was identified and corrected, the newly revised CT plan did not differ from its corresponding syCT.

**Figure 3:**
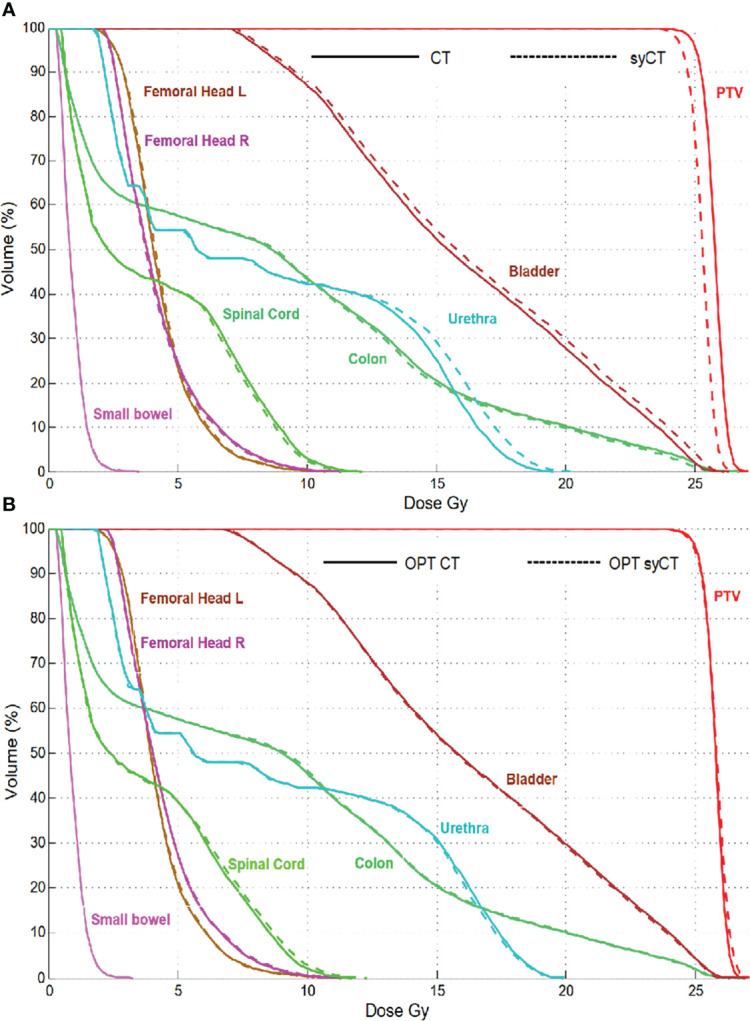
Rectum case 1: DVH comparison of the original CT plan and its corresponding syCT plan **(A)** and of the reworked CT reference plan with its corresponding syCT **(B)**.

Rectum case 1, the first rectum treatment scheduled at MR-Linac, was immobilized with a thermoplastic mask clamped to the patient positioning system (pps) plate as for standard Linacs. The isodoses of the reference CT plan differed significantly from the corresponding ones of syCT; moreover, no dose was visible within pps plate in the CT, whereas it was displayed on syCT. Further investigation established that, for the pps to be included in the TPS calculation, it is necessary to add such a region in the within the optimisation parameters. After reworking the reference plan, the doses obtained on syCT matched the doses calculated on the reference CT plan, as shown by the DVHs in **Figure 3**. For the rectum case 2, we observed an inappropriate setting in the layer prioritization of the parts of the pps plate with different ED, which was also corrected to achieve an agreement between CT and syCT dosimetry.

**Figure 4:**
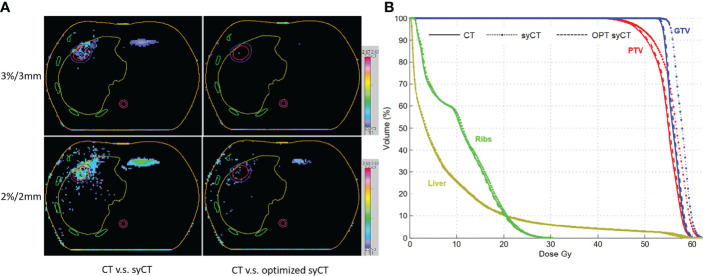
The gamma analysis among CT, syCT, and optimized syCT with criteria of 3%/3 mm and 2%/2 mm, respectively **(A)**; the comparison of DVH among CT, syCT, and optimized syCT **(B)**.

Regarding the CT and syCT dose discrepancy of the brain treatment, in the original CT plan, the region of interest (ROI) of the whole brain was given priority in ED allocation over other ROIs, resulting in the loss of skull on the syCT and leading to an unavoidable dosimetric difference. For the two liver cases, the lung and target contours drawn on the MIP CT did not match the organs on the reference CT, thus generating an incorrect ED assignment on the syCT and leading to an understandable dose difference in both DVH dosimetry and gamma analysis. **Figure 4** shows, for liver case 1, the gamma analysis results between the dose distribution of CT and syCT in transverse plane, before and after the original CT plan reworking, and corresponding DVHs.

## Discussion

The use of MRgRT has raised the quality of all those treatments for which soft tissue visualization is essential, particularly suitable for precision treatments when the targets are close to risky structures and even more when high fractional doses are to be delivered (4, 7, 9, 53, 54). MRgRT performed with the Unity Linac allows through the ATS workflow to recalculate and reoptimize the plan using a syCT generated from the daily MRI. It remains therefore a crucial point to put in place all possible actions to ensure the accuracy of the calculation on this MR-based syCT (49).

We have included in the clinical workflow a procedure to assess, for each individual treatment plan, the minimum error that we should expect when performing the recalculation on the syCT in the ATS procedure. The procedure consists of generating an ideal syCT using the reference CT and the information included in the reference CT plan, recalculating the original plan on the syCT, and comparing the dosimetry of the original CT and syCT with the DVHs. It is therefore a very intuitive procedure, easy to perform, and with short execution times; for the purpose of the research work, we added the gamma analysis evaluation, which gives further confirmation of the agreement/disagreement between the two dose distributions.

The results were satisfactory for most of the patients (54 out of 61). For the patients who had a syCT plan discordant with the original CT plan, the procedure allowed us to highlight the following: i) incorrect information or input given to the patient’s treatment plan—treatment plan that was then reworked before its clinical use; ii) errors in the clinical workflow—revised and optimized; and iii) discrepancies related to tumor morphology/location not manageable by the system—patients treated using another procedure. It is important to point out that these errors could not have been detected by the pre-treatment checks or at the plan adaptation phase. Moreover, the well-known criticalities in the treatment of targets in areas of high inhomogeneity (lung targets) can be quantified in a very simple way through the discordance of DVHs.

One of the criticisms of the ATS procedure is certainly the fact that the contours on simulation CT are automatically propagated by deformable registration onto the online planning MRI and then edited by a radiation oncologist; this step has repercussions on the adequacy the MRI-based syCT plan calculation, as each organ is assigned an average ED; therefore, the correctness of the target contour and the OAR identification as well as the choice of the significant number of contours are of paramount importance. Regarding the adjustment of OARs, if the constraints are related to the maximum dose, although it might seem reasonable to touch up only the part of the organ adjacent to the target, because the organ has its own density, then it is necessary for the accuracy of the calculation to edit it in full. Similarly, the ribs, which for most treatments are not drawn, in the case of recalculation on the syCT, are necessary to outline at least those that intersect the beams entry. Auto-segmentation could be a feasible solution to automatically contour targets and OARs during the online adaptive phase (55). Furthermore, considering that the calculation on the syCT is governed not only by the shape of the drawn organs but also by their average ED, it must be ensured that the priority of one organ over the other in case of their overlapping has been correctly assigned and that, in any case in the chain of successive steps, there has not been a “disturbing element” that could have influenced the final dose delivered. Organs with different densities can be an issue for the calculation accuracy; consequently, it would therefore be completely inappropriate to force a target to have an average ED if it includes parts with different densities.

The use of our procedure within the clinical workflow could be seen as an additional control to be included in the quality assurance of the treatment. Considering the sensitivity of the system to inhomogeneities, the organs that may have different densities within them (air bubbles in the case of the intestine, PTV including different tissues) must be properly managed within a clinical workflow; moreover, we must consider that the manual modification of the contours must anyway be done in a reasonable time. Whenever the MRI scan acquired at simulation would be used as imaging for the reference plan, the procedure proposed may also be useful; in this case, the transport of the contours between MR images (simulation to daily MR) should be more accurate, even if we cannot avoid their verification/editing if deemed necessary (48). Moreover, this process could be carried out off-line before the patient’s treatment begins and would therefore have robustness comparable to the recalculation on the planning CT.

Among the limitations of this procedure is the fact that it only allows the estimation of the minimum error introduced by the bulk density assignment–based syCT. This therefore does not exclude unmanageable errors during the online process, as not accurate or exhaustive recontouring of OARs and all regions supposed to have different ED. Furthermore, high-density parts of a pps within the treatment field can affect dosimetry. In fact, no matter how accurately the patient may be positioned, small movements of the patient within the thermoplastic mask are always possible. Even if the parts of the pps in question were contoured during the treatment plan, the MRI would not be able to visualise them during treatment, and therefore, we would not be able to adjust for small differences as we do with contours. The recalculation on the online procedure would unfortunately not take this into account.

## Conclusion

The accuracy of the reference treatment plan calculation on a SyCT can be influenced by several factors in the different steps of the clinical workflow of the Unity MR-Linac. The evaluation of the accuracy of its calculation can be easily inserted in the radiotherapy routine of the individual patient to highlight and correct in time the errors that may occur and to provide the RO with guidance on proceeding with treatment using either ATS or ATP.

## Data availability statement

The raw data supporting the conclusions of this article will be made available by the authors, without undue reservation.

## Ethics statement

The studies involving human participants were reviewed and approved by Ethics Committee of Sichuan Cancer Hospital, Sichuan Cancer Hospital. The patients/participants provided their written informed consent to participate in this study.

## Author contributions

Conceptualization: BT and LO. Data acquisition: ML, PD, FW, XL, XF, and XY. Data analysis: ML, BW, FW, XF, and QH. Project administration: JL. Writing—original draft: BT and LO. Supervision: JL, LO, BT, and QH. Writing—review and editing: BT, BW, PD, FW, and XY. All authors contributed to the article and approved the submitted version.

## Funding

This research was supported by Deng Feng Precision Radiotherapy Research Program (2021DF016) and Sichuan Province Science and Technology Support Program (2022JDGD0032).

## Acknowledgments

We would like to thank Sichuan Guo from Elekta Company for his technical support on our clinical work.

## Conflict of interest

The authors declare that the research was conducted in the absence of any commercial or financial relationships that could be construed as a potential conflict of interest.

## Publisher’s note

All claims expressed in this article are solely those of the authors and do not necessarily represent those of their affiliated organizations, or those of the publisher, the editors and the reviewers. Any product that may be evaluated in this article, or claim that may be made by its manufacturer, is not guaranteed or endorsed by the publisher.
